# 112. A Rapid Host-Protein Signature Based on TNF-related Apoptosis-Induced Ligand (TRAIL), Interferon Gamma Induced Protein-10 (IP-10) and C-Reactive Protein (CRP) Accurately Differentiates Between Bacterial and Viral Infection in Febrile Children: Apollo Sub-Study

**DOI:** 10.1093/ofid/ofab466.112

**Published:** 2021-12-04

**Authors:** Sheldon L Kaplan, Adi Klein, Mark Kellogg, Andrea T Cruz, Kristina G Hulten, Cesar A Arias, Richard Gordon, Sergey Motov, Theresa Jacob, Natasha Ballard, George Suits, Jeffrey Harris, Maanit Shapira, Richard E Rothman, Karen C Carroll, Karen C Carroll, Leticia M Ryan, Richard Bachur

**Affiliations:** 1 Baylor College of Medicine, Houston, TX; 2 Hillel Yaffe Medical Center, Hadera, HaMerkaz, Israel; 3 Boston Children's Hospital, Boston, Massachusetts; 5 CARMiG, UTHealth and Center for Infectious Diseases, UTHealth School of Public Health, HOU, TX ; Molecular Genetics and Antimicrobial Resistance Unit and International Center for Microbial Genomics, Universidad El Bosque, BOG, COL, Houston, Texas; 6 University Texas Health Science Center, Houston, Texas; 7 Maimonides Medical Center, New York, New York; 8 AFC Urgent Care, Chattanooga, Tennessee; 9 Urgent care center, Greenville, South Carolina; 10 Johns Hopkins University School of Medicine, Baltimore, MD; 11 Johns Hopkins University, Baltimore, Maryland

## Abstract

**Background:**

Identifying infectious etiology is essential for appropriate patient management, including antibiotic use. A host-protein signature for differentiating bacterial from viral infection has exhibited robust performance (AUC of 0.9, 95% CI 0.86-0.95) in prior studies. Performance data was lacking for a broad pediatric population recruited in emergency departments (EDs) and urgent care centers (UCCs).

**Methods:**

Non-immunocompromised children were recruited prospectively from 5 EDs and 3 UCCs in the U.S. and 1 ED in Israel between May 2019 and August 2020. Eligibility required physician’s clinical suspicion of acute infection and reported fever. Reference standard etiology was adjudicated by experts based on clinical, laboratory, radiological, microbiological and follow-up data. For the primary analysis, experts blinded to one another, to the host-signature results and also to procalcitonin and CRP, classified cases as bacterial or viral. For the secondary analysis, experts blinded to one another and the host signature results, were permitted to classify cases as bacterial, viral or indeterminate; indeterminates were removed from the secondary analysis. Host signature (comprising TRAIL, IP-10 and CRP; MeMed BV®) was measured using a rapid platform (MeMed Key®) generating a bacterial likelihood score (0-100) in 15 minutes.

**Results:**

The study cohort comprised 162 children (median age, 5.5 yrs; interquartile range, 8.5), of whom 69 (43%) presented within 2 days of symptom onset and 37 (23%) were hospitalized for a median of 3 days. Respiratory tract infection was the predominant syndrome (11% lower and 44% upper). Host signature attained AUC 0.87 (0.74-1) and 0.92 (0.79-1) in the primary and secondary analysis, respectively. With higher the signature score, there was a significantly higher likelihood of bacterial infection (p< 0.001; Table 1). The 3 bacterial infections assigned score < 35 (false negative) would have been identifiable by physical examination (Table 2).

Increasing host signature score is associated with increasing likelihood of bacterial infection across both the primary and secondary cohort

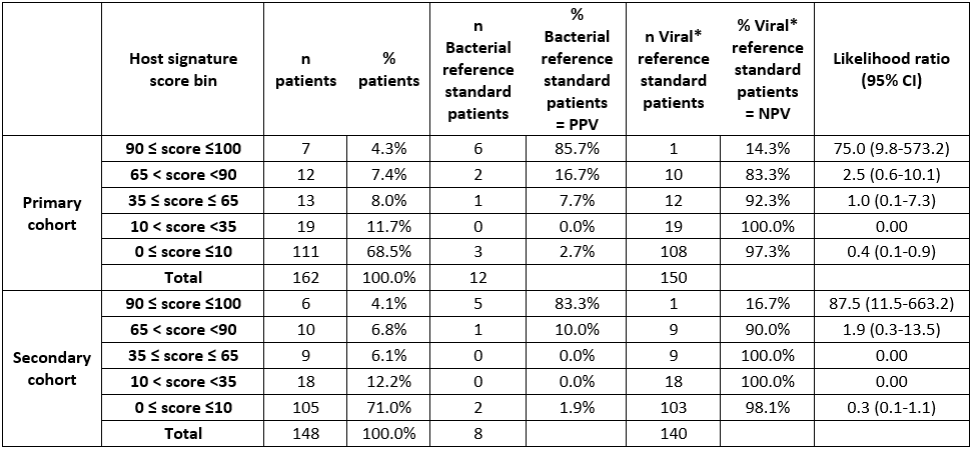

The performance of the host signature score in differentiating between bacterial and viral infection was evaluated by allocating children to one of five score bins and within each bin according to their adjudication label and determining if there is a meaningful increase in the relative likelihood of bacterial infection across the bins based on the Cochrane-Armitage test of trend. PPV, positive predictive value. NPV, negative predictive value. *Includes patients adjudicated as non-infectious

Three children assigned a bacterial adjudication label and a score of 35 or less (false negatives) have bacterial infections identifiable in physical exam

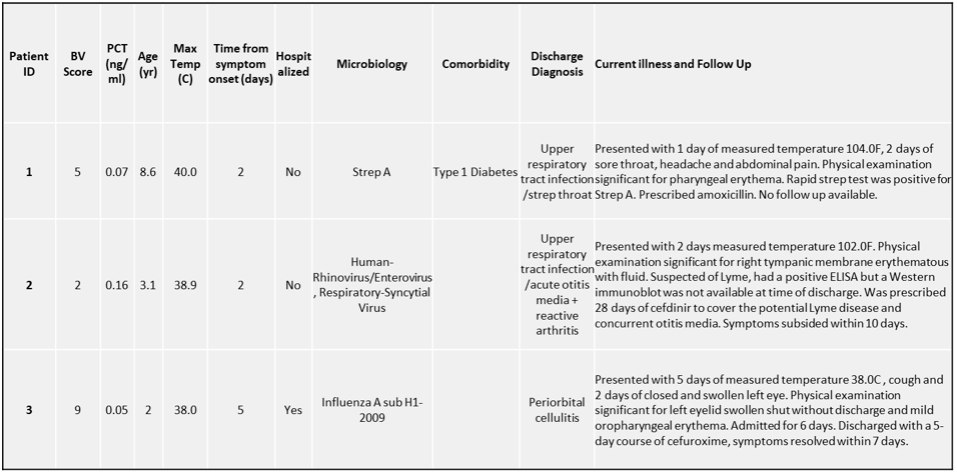

**Conclusion:**

The host-protein signature measured using a rapid platform attained robust performance in differentiating bacterial vs viral infection in children with acute febrile illness, supporting its potential to enhance rational use of antibiotics in the ED and UCC.

**Disclosures:**

**Sheldon L. Kaplan, MD**, **Pfizer** (Research Grant or Support) **Mark Kellogg, PhD**, **MeMed** (Scientific Research Study Investigator) **Andrea T. Cruz, MD, MPH**, American Academy of Pediatrics (Individual(s) Involved: Self): editorial board member **Kristina G. Hulten, PhD**, **Pfizer** (Research Grant or Support) **Cesar A. Arias, M.D., MSc, Ph.D., FIDSA**, **Entasis Therapeutics** (Grant/Research Support)**MeMed Diagnostics** (Grant/Research Support)**Merk** (Grant/Research Support) **Richard Gordon, MD**, **MeMed** (Scientific Research Study Investigator) **Sergey Motov, MD**, **MeMed** (Scientific Research Study Investigator) **Theresa Jacob, PHD MPH**, **MeMed** (Scientific Research Study Investigator) **Natasha Ballard, MD**, **MeMed** (Scientific Research Study Investigator) **George Suits, MD**, **MeMed** (Scientific Research Study Investigator) **Jeffrey Harris, MD**, **MeMed** (Scientific Research Study Investigator) **Maanit Shapira, Ph.D**, **MeMed** (Scientific Research Study Investigator) **Richard E. Rothman, PhD, MD**, **Chem bio** (Grant/Research Support) **Karen C. Carroll, MD**, **MeMed** (Scientific Research Study Investigator)**Meridian Diagnostics, Inc.** (Grant/Research Support)**Pattern Diagnostics** (Advisor or Review Panel member)**Scanogen, Inc.** (Advisor or Review Panel member) **Karen C. Carroll, MD**, Pattern Diagnostics, Inc. (Individual(s) Involved: Self): Grant/Research Support; Scanogen, Inc. (Individual(s) Involved: Self): Consultant **Leticia M. Ryan, MD MPH**, **MeMed** (Scientific Research Study Investigator) **Richard Bachur, MD**, **MeMed** (Scientific Research Study Investigator)

